# Thyroxine modifies the effects of growth hormone in Ames dwarf mice

**DOI:** 10.18632/aging.100739

**Published:** 2015-04-22

**Authors:** Andrew Do, Vinal Menon, Xu Zhi, Adam Gesing, Denise S. Wiesenborn, Adam Spong, Liou Sun, Andrzej Bartke, Michal M. Masternak

**Affiliations:** ^1^ Burnett School of Biomedical Sciences, College of Medicine, University of Central Florida, Orlando, FL 32827, USA; ^2^ Department of Cell Biology and Anatomy, School of Medicine, University of South Carolina Columbia, SC 29209, USA; ^3^ Center of Reproductive Medicine, Department of Obstetrics and Gynecology, Peking University Third Hospital, Beijing 100191, China; ^4^ Department of Oncological Endocrinology, Medical University of Lodz, 90‐752 Lodz, Poland; ^5^ Department of Medical Biochemistry and Molecular Biology, University of Saarland, 66421 Homburg, Germany; ^6^ Department of Biotechnology, University of Applied Sciences Kaiserslautern, 66482 Zweibrücken, Germany; ^7^ Department of Internal Medicine, Southern Illinois University School of Medicine, Springfield, IL 62794, USA; ^8^ Department of Head and Neck Surgery, The Greater Poland Cancer Centre, 61‐866 Poznan, Poland

## Abstract

Ames dwarf (df/df) mice lack growth hormone (GH), thyroid stimulating hormone and prolactin. Treatment of juvenile df/df mice with GH alone stimulates somatic growth, reduces insulin sensitivity and shortens lifespan. Early‐life treatment with thyroxine (T4) alone produces modest growth stimulation but does not affect longevity. In this study, we examined the effects of treatment of juvenile Ames dwarf mice with a combination of GH + T4 and compared them to the effects of GH alone. Treatment of female and male dwarfs with GH + T4 between the ages of 2 and 8 weeks rescued somatic growth yet did not reduce lifespan to match normal controls, thus contrasting with the previously reported effects of GH alone. While the male dwarf GH + T4 treatment group had no significant effect on lifespan, the female dwarfs undergoing treatment showed a decrease in maximal longevity. Expression of genes related to GH and insulin signaling in the skeletal muscle and white adipose tissue (WAT) of female dwarfs was differentially affected by treatment with GH + T4 vs. GH alone. Differences in the effects of GH + T4 vs. GH alone on insulin target tissues may contribute to the differential effects of these treatments on longevity.

## INTRODUCTION

Ames dwarf mice (df/df) have underdeveloped anterior pituitary glands due to a homozygous recessive deletion at the Prop1 locus. This loss of function mutation leads to primary hormonal deficiencies in growth hormone (GH), thyroid stimulating hormone (TSH), and prolactin (PRL) [1-3]. As a result, Ames dwarf mice have secondary deficiencies in insulin-like growth factor-1(IGF-1) and thyroid hormones (T4). Furthermore, df/df mice have reduced circulating levels of insulin and glucose. This implies enhanced insulin sensitivity, a conclusion which is supported by glucose tolerance and insulin tolerance tests, as well as by a recent study involving hyperinsulinemic-euglycemic clamps [4-7]. Importantly, Ames dwarf mice show a delayed aging process, demonstrated by enhancements in lifespan and healthspan including maintenance of higher insulin sensitivity and glucose tolerance throughout life, preservation of cognitive and neuromuscular function, and decreased occurrence of cancer [6, 8-12]. Mechanisms of the 40-60% increase in lifespan of df/df mice most likely include the interruption in somatotropic (GH/IGF-1) signaling and enhanced insulin sensitivity [5, 13, 14], along with enhancement of anti-oxidant defenses and stress resistance [15-20]. Numerous studies in several animal models reinforce the correlations between insulin sensitivity and the effects of GH on longevity. The dietary intervention of calorie restriction (CR) is a reduction in total calories consumed; it produces an increase in insulin sensitivity, lifespan, and healthspan in many animal species, including mice [4, 21-23]. While both df/df mice and CR mice show positive signs of healthier aging, they appear to do so via different mechanisms, considering that df/df mice undergoing 30% CR exhibit a further extension of longevity [1, 4, 21, 24, 25]. Furthermore, Ames, Snell, and Laron dwarf mice all feature disruptions in the GH/IGF-1 axis and have reduced plasma concentrations of glucose and insulin, as well as increases in lifespan and healthspan when compared to normal littermates [8, 26].

Insulin sensitivity is also related to aging and longevity in humans. Thus, glucose tolerance tends to decline with age and approximately 27% of the elderly over 65 are being diagnosed with type 2 diabetes [27, 28]. In contrast, populations of centenarians have been shown to have improved insulin action, increased adiponectin, and either a reduction in serum IGF-1 levels or a higher prevalence of functional IGF-1 receptor mutations [29-34]. There is a marked progressive decline in GH levels that begin after puberty [35, 36]. However, centenarians have not been shown to have significantly different levels of serum GH compared to normal, healthy aged individuals [37]. On the other hand, overexpression of GH is associated with detrimental effects on health in both humans and mice, including tumor development, insulin resistance, reduced antioxidant activity, reduced immune function, and shorter lifespan [38-41].

Treatment of juvenile male Ames dwarf mice with GH markedly increases somatic growth, but severely attenuates insulin sensitivity, glucose tolerance, cellular stress resistance, and longevity [5, 6, 42]. After GH treatment is discontinued, somatic growth slows down and body weight stabilizes at a level intermediate between normal (wild-type) and untreated Ames dwarf mice, while insulin sensitivity reduced by GH therapy is eventually restored [5]. The ability of early-life, six week GH treatment to reduce the longevity of Ames dwarf mice applies to both females and males and is reproducible (Hill, Arum and Bartke, unpublished observations). Juvenile male and female Ames dwarf mice treated for six weeks with thyroxine (T4) experienced increases in bodyweight, yet longevity was not significantly affected [6]. Young male Ames dwarf mice treated with a combination of GH and T4 exhibited a major increase in bodyweight, approaching the body weight of their normal siblings. The treatment with GH plus T4 also led to increased plasma insulin, decreased adiponectin, and decreased expression of insulin signaling components in the liver [43].

In the present study, we have examined the impact of six weeks of treatment of juvenile Ames dwarf mice with a combination of GH and T4 on their longevity. We also analyzed the effects of treating female Ames dwarf mice with GH alone or with GH plus T4 on the expression of genes involved in insulin signaling in skeletal muscle (SM) and white adipose tissue (WAT). To determine the effects of the hormonal intervention we performed an analysis of the expression of genes involved in the insulin signaling pathway, including insulin receptor (IR), insulin receptor substrate-1 (IRS-1), and phosphatidylinositol bisphosphate 3-kinase (PI3K). Next, we investigated an important fulcrum in insulin signaling pathway, the serine/threonine kinase Akt2, as well as genes of its downstream effectors, namely GLUT4 and forkhead box O3 (FOXO3). The mRNA levels of the transcription factor peroxisome proliferator-activated receptor gamma (PPAR-γ), peroxisome proliferator-activated receptor gamma coactivator 1 alpha (PGC-1β), and Adiponectin (an adipokine) were examined, as they are important modulators of insulin sensitivity, glucose utilization, and lipid metabolism. Additionally, to determine the activation of GH signals in df/df mice after the treatment, we examined the expression of the growth hormone receptor (GHR) gene and genes related to its downstream effects, including insulin-like growth factor-1 (IGF-1), STAT-1, STAT-5a, and STAT-5b.

## RESULTS

### Longevity

**Figure 1:**
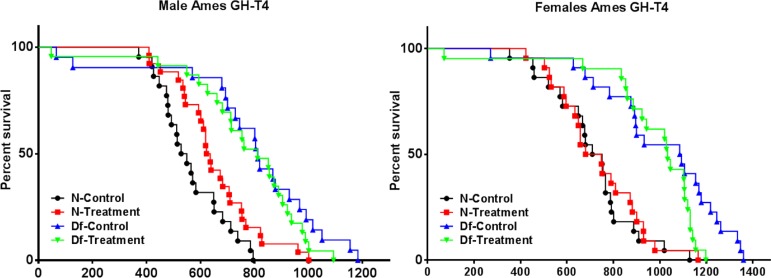
Longevity study Male and Female Kaplan‐Meier survival plots of Normal (N) and Ames dwarf (df/df) mice with either no treatment (saline) or treated with a combination of growth hormone and thyroxine (GH + T4) (n = 24 animals per group).

Consistent with our previous reports [4, 6, 18], Ames dwarf mice lived significantly longer than normal animals (p < 0.0001; log-rank test). Average longevity of female and male Ames dwarf mice that had been treated with GH + T4 between 2 and 8 weeks of age did not differ from the longevity of dwarf mice of the same sex that had been injected with saline. We used the Wang/Allison method to compare the proportion of live mice in each group at the age at which only 10% of the population remained alive [44]. Maximal longevity of female (p = 0.0128 by Fisher's exact test), but not male Ames dwarf mice, was reduced by GH + T4 treatment. Unexpectedly, treatment of normal juvenile male (but not female) mice with GH + T4 for 6 weeks produced a small but statistically (p = 0.0360; log-rank test) significant increase in overall longevity (Figure 1).

### Body weight and Glucose Level

**Figure 2:**
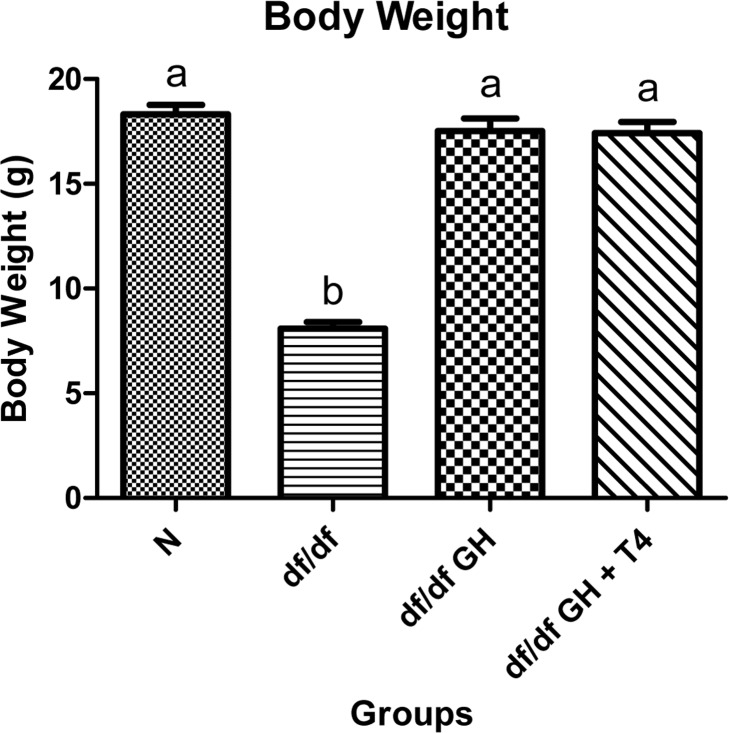
Body weight comparison of 8 week old female Normal mice (N, n=10), Ames dwarf mice (df/df, n=10), Ames dwarf mice treated with GH (df/df GH, n=10), and Ames dwarf mice treated with both GH and T4 (df/df GH + T4, n=8). Groups that do not share a superscript are different with statistical significance (p < 0.05).

At the age of 2 months, body weight of the Ames dwarf mice was significantly lower than phenotypically normal (N) control mice as expected (p < 0.0001). However, after six weeks of treatment with GH or GH and T4, the df/df mice were significantly larger than saline treated mutants (p < 0.0001) and matched the body weight of normal mice (Figure 2).

**Figure 3:**
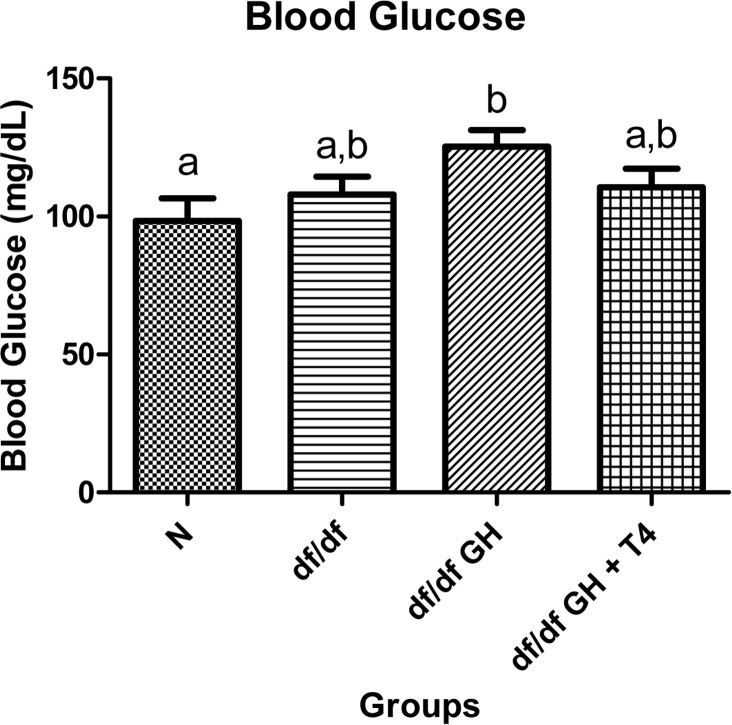
Blood glucose comparison of 8 week old female Normal mice (N, n=10), Ames dwarf mice (df/df, n=10), Ames dwarf mice treated with GH (df/df GH, n=10), and Ames dwarf mice treated with both GH and T4 (df/df GH + T4, n=8). Groups that do not share a superscript are different with statistical significance (p < 0.05).

Female df/df-GH mice showed a numerical increase of fasting blood glucose levels reaching values higher than the levels measured in the normal mice (p < 0.05). However, dwarf mice treated with GH and T4 showed no such increase with fasting blood glucose levels resembling those in untreated df/df mice (Figure 3).

### Gene Expression: Skeletal Muscle

**Figure 4:**
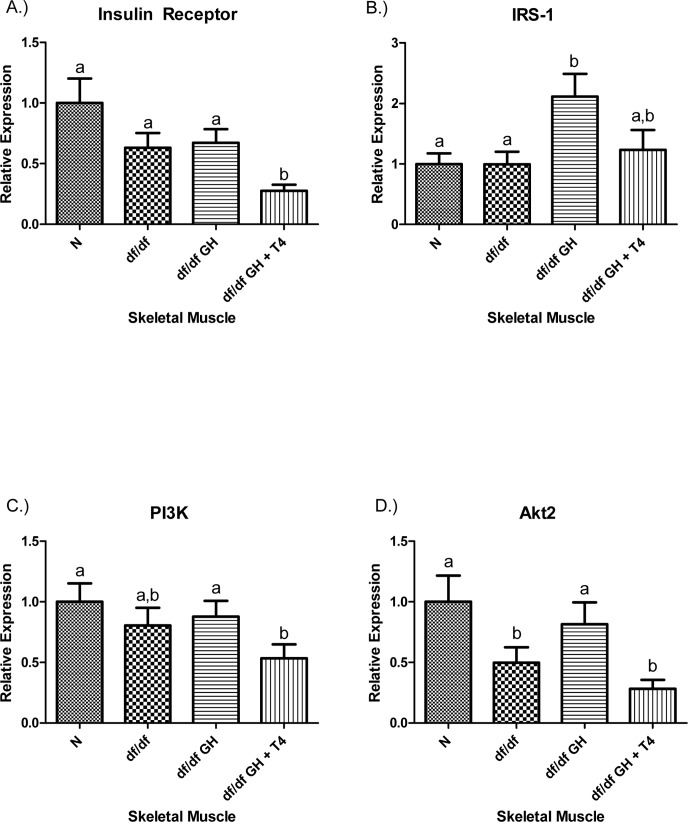
(**A**) Insulin Receptor (IR), (**B**) Insulin Receptor Substrate‐1 (IRS‐1), (**C**) Phosphoinositide 3‐Kinase (PI3K), and (**D**) Akt2 relative gene expression in female Ames dwarf (df/df) mouse skeletal muscle tissue after growth hormone (GH) and thyroxine (T4) treatment. For A‐D, Normal (N) n = 10; df/df n = 10; df/df GH n = 10; and df/df GH + T4 n = 8. Groups that do not share a superscript show differences with statistical significance (p < 0.05).

There were no differences in insulin receptor (IR) gene expression in the skeletal muscle tissue between N, df/df and df/df-GH mice, while df/df-GH + T4 mice had reduced IR mRNA expression (p < 0.01). IRS-1 gene expression was significantly increased in df/df-GH mice when compared with df/df animals (p < 0.01), yet in the group given GH plus T4, this effect of GH was attenuated. PI3K gene expression was not altered by Ames dwarfism or treatment with GH alone. However, combined treatment with GH + T4 significantly reduced PI3K mRNA expression in comparison to both N mice and mice treated with GH alone (p < 0.01) (Figure 4). The expression level of skeletal muscle Akt2 was decreased in df/df mice when compared to N mice (p < 0.05). GH treatment normalized the expression of Akt2, while GH-T4 treatment did not with no significant difference from values measured in untreated df/df mice (p < 0.05) (Figure 4).

**Figure 5:**
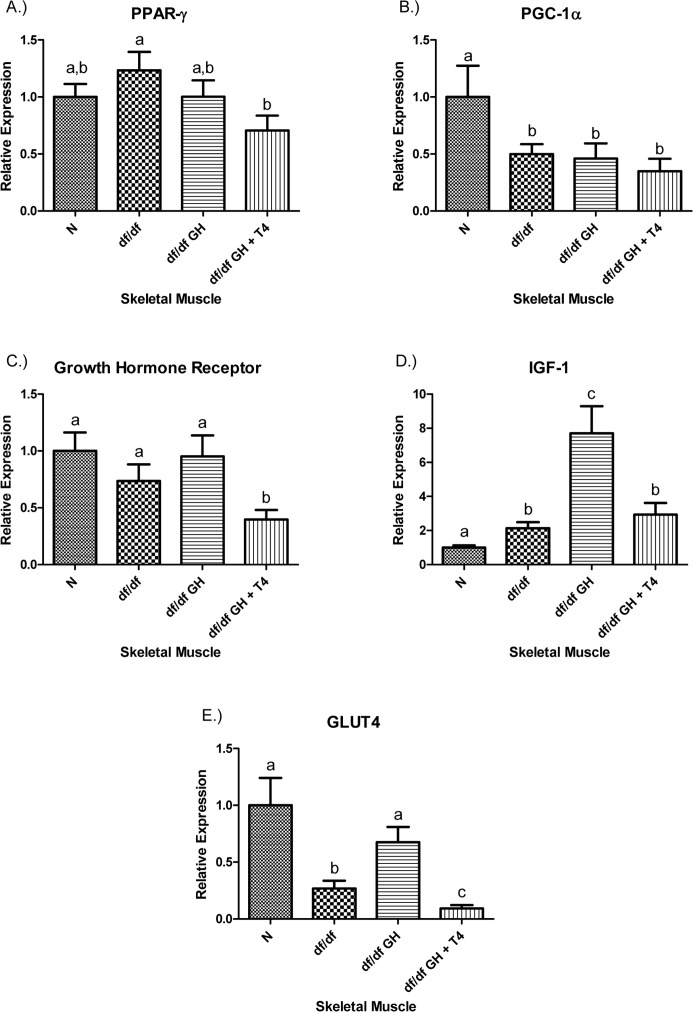
(**A**) Peroxisome Proliferator‐Activated Receptor (PPAR) γ, (**B**) PPAR Coactivator 1α (PGC‐1α), (**C**) Growth Hormone Receptor, (**D**) Insulin‐like Growth Factor‐1 (IGF‐1), and (**E**.) GLUT4 relative gene expression in female Ames dwarf (df/df) mouse skeletal muscle tissue after growth hormone (GH) and thyroxine (T4) treatment. For A‐E, Normal (N) n = 10; df/df n = 10; df/df GH n = 10; and df/df GH + T4 n = 8. Groups that do not share a superscript show differences with statistical significance (p < 0.05).

The level of PPAR-γ mRNA in skeletal muscle was not altered in df/df mice and not affected by GH therapy. However, combined GH-T4 treatment significantly reduced the PPAR-γ mRNA expression (p < 0.05) (Figure 5). The expression level of PGC-1α was decreased in all groups of Ames dwarf mice, regardless of the treatment (p < 0.05). The growth hormone receptor (GHR) mRNA level was not altered in GH-deficient df/df mice and was not affected by GH. Interestingly, df/df-GH + T4 mice showed a decrease in the expression level of GHR when compared with either N (p< 0.005), df/df (p < 0.05), or df/df-GH animals (p < 0.05) (Figure 5). The IGF-1 mRNA level in the skeletal muscle was significantly increased in df/df mice when compared to N animals (p < 0.005), and GH treatment caused a further increase of local IGF-1 expression (when compared with either N or df/df mice; p < 0.0005 and p < 0.005, respectively). Interestingly, addition of T4 attenuated the effect of GH treatment and maintained the expression of IGF-1 mRNA in df/df-GH + T4 at the level observed in df/df control mice (Figure 5). In comparison to N animals, control Ames dwarf mice had markedly reduced GLUT4 gene expression in (p < 0.005), and treating them with GH alone increased GLUT4 gene expression to the levels found in normal mice (p < 0.01). In contrast, treatment with GH plus T4 suppressed GLUT4 gene expression to levels below those measured in the df/df and df/df-GH groups (p < 0.05, p < 0.001 respectively) (Figure 5).

**Figure 6:**
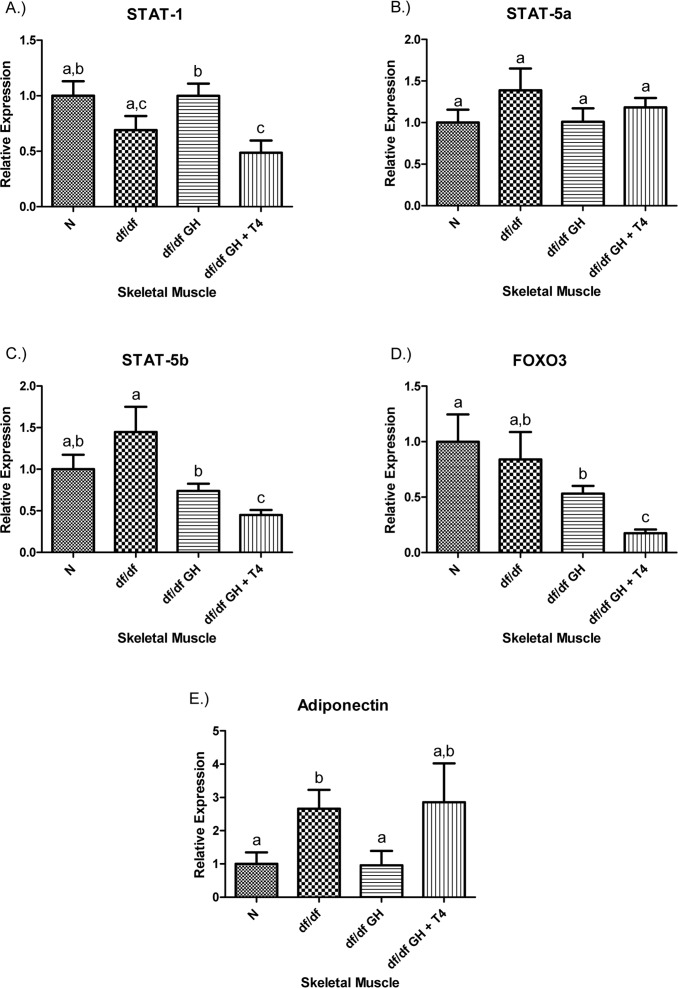
(**A**) Signal Transducer and Activator of Transcription (STAT) ‐1, (**B**) STAT‐5a, (**C**) STAT‐5b, (**D**) Adiponectin, and (**E**) FOXO3 relative gene expression in female Ames dwarf (df/df) mouse skeletal muscle tissue after growth hormone (GH) and thyroxine (T4) treatment. For A‐E, Normal (N) n = 10; df/df n = 10; df/df GH n = 10; and df/df GH + T4 n = 8. Groups that do not share a superscript show differences with statistical significance (p < 0.05).

Growth hormone treatment of dwarf mice elevated STAT-1 gene expression (p < 0.05), but combined GH and T4 therapy did not. STAT-1 expression in animals treated with GH + T4 mice was significantly lower than in the group given GH alone (p < 0.005). The STAT-5a expression was not affected by genotype or by either of the treatments (Figure 6). However, STAT-5b expression was reduced by GH treatment of df/df mice (p < 0.05), and GH-T4 treatment led to a further significant decrease in the expression of this gene (p < 0.01). The expression of FOXO3 transcription factor was decreased in df/df-GH + T4 group in comparison to N, df/df and df/df-GH animals (P < 0.005, p < 0.05, and p < 0.0005, respectively). The markedly increased adiponectin mRNA levels in untreated df/df mice (p < 0.05), were normalized by GH treatment (p < 0.05) but were not affected by administration of GH together with T4 (Figure 6).

### Gene Expression: White Adipose Tissue

**Figure 7:**
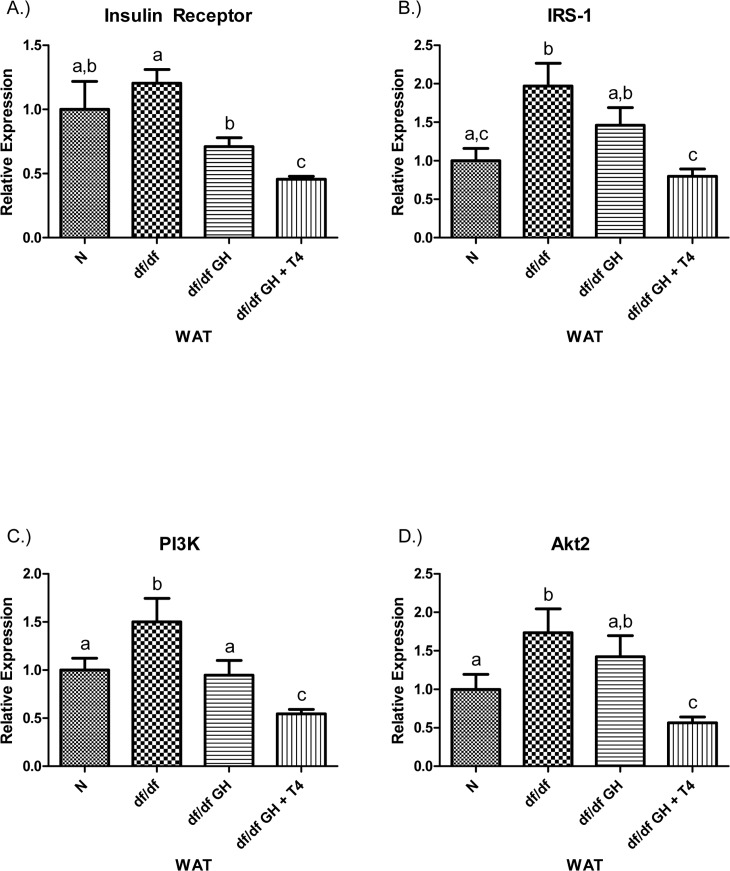
(**A**) Insulin Receptor (IR), (**B**) Insulin Receptor Substrate‐1 (IRS‐1), (**C**) Phosphoinositide 3‐Kinase (PI3K), and (**D**) Akt2 relative gene expression in female Ames dwarf (df/df) mouse white adipose tissue after growth hormone (GH) and thyroxine (T4) treatment. For A‐D, Normal (N) n = 10; df/df n = 9; df/df GH n = 9; and df/df GH + T4 n = 8. Groups that do not share a superscript show differences with statistical significance (p < 0.05).

The level of IR mRNA in white adipose tissue (WAT) did not differ between N and df/df mice. Growth hormone treatment suppressed IR mRNA expression in dwarf mice (p < 0.001) with further significant decrease in animals given GH + T4 (p < 0.005). Interestingly, IR expression in df/df-GH + T4 animals was significantly lower than in N mice (p < 0.05). The IRS-1 mRNA expression level was increased in df/df mice when compared with N animals (p < 0.005), and normalized by treatment with GH and T4 (p < 0.005), but not with GH alone (Figure 7). PI3K mRNA expression was elevated in df/df mice when compared to N littermates (p < 0.05). GH treatment normalized PI3K expression (p < 0.05), while GH plus T4 treatment led to a further suppression of the levels of PI3K mRNA (when compared with N, df/df and df/df-GH groups, p < 0.005, p < 0.005, p < 0.05, respectively). The level of Akt2 mRNA was up-regulated in df/df when compared to N mice (p < 0.05), not affected by treatment with GH alone but reduced by treatment with GH + T4 to levels significantly below those measured in the df/df an df/df-GH groups (p < 0.005 and p < 0.01, respectively) (Figure 7).

**Figure 8:**
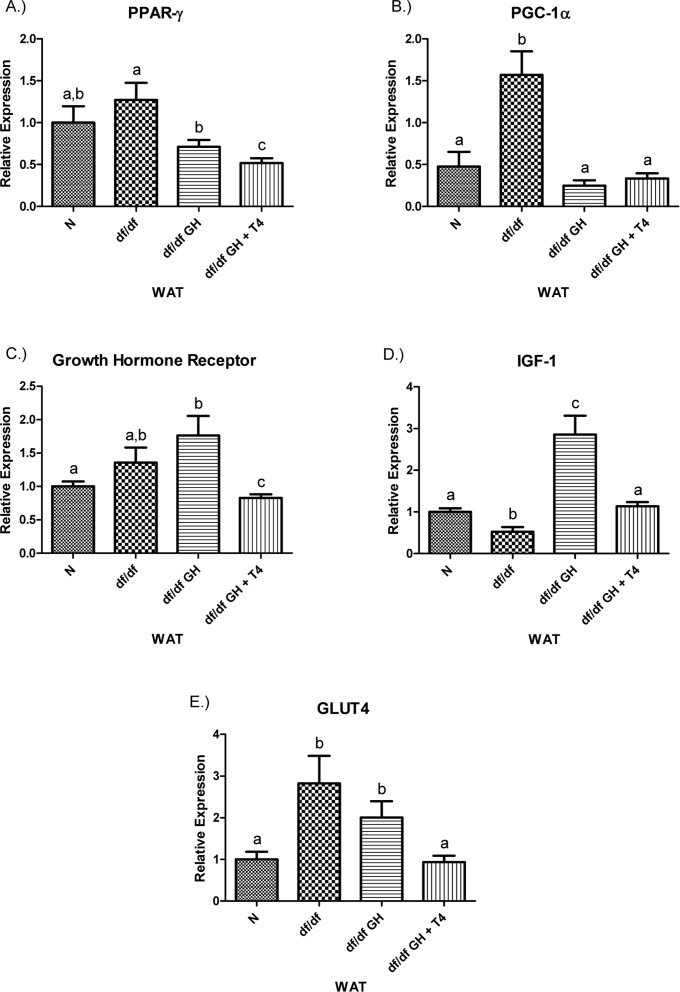
(**A**) Peroxisome Proliferator‐Activated Receptor (PPAR) γ,(**B**) PPAR Coactivator 1α (PGC‐1α), (**C**) Growth Hormone Receptor, (**D**) Insulin‐like Growth Factor‐1 (IGF‐1), and (**E**) GLUT4 relative gene expression in female Ames dwarf (df/df) mouse white adipose tissue after growth hormone (GH) and thyroxine (T4) treatment. For A‐E, Normal(N) n =10; df/dfn=9; df/dfGHn = 9; and df/dfGH+ T4 n = 8. Groups that do not share a superscript show differences with statistical significance (p < 0.05).

Both GH and GH + T4 treatment suppressed PGC-1α mRNA in df/df-GH and df/df-GH + T4 mice to the levels maintained by N mice (p < 0.0001, p < 0.0005 respectively). The level of GHR mRNA was not altered in untreated df/df mice and treatment with GH increased it to a level higher than in N animals (p < 0.01). Interestingly, the combined GH plusT4 therapy caused a decrease inGHR gene expression, when compared with N, df/df, and df/df-GH mice (p < 0.05, p < 0.05and p < 0.005, respectively). The expression of IGF-1 in the adipose tissue of Ames dwarf was reduced (p < 0.005). GH treatment increased IGF-1 mRNA levels in comparison with N and df/df littermates (p < 0.0005 and p < 0.0001, respectively); while, combined treatment with GH and T4 had a much smaller effect leading to normalization of IGF-1 expression. Ames dwarf controls showed increased expression of the GLUT4 gene vs. N mice (p < 0.01). While GH treatment had no significant effect on the expression of this gene, treatment with GH plus T4 significantly decreased GLUT4 mRNA levels when compared with df/df and df/df-GH mice (p < 0.001 and p < 0.005, respectively), normalizing it to the level observed in N animals (Figure 8).

**Figure 9:**
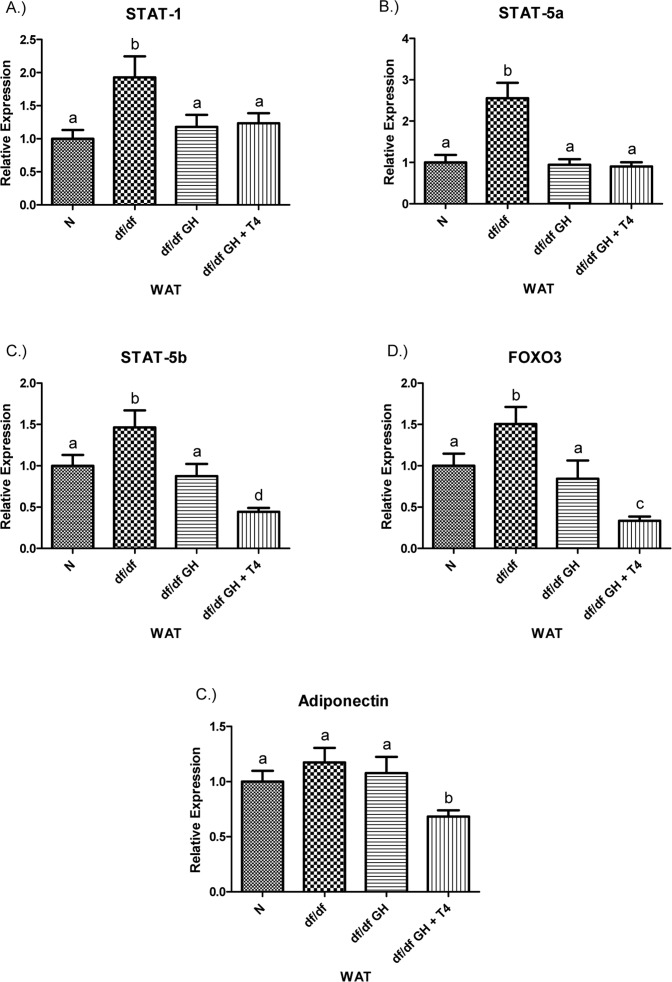
(**A**) Signal Transducer and Activator of Transcription (STAT) ‐1, (**B**) STAT‐5a, (**C**) STAT‐5b, (**D**) Adiponectin, and (**E**) FOXO3 relative gene expression in female Ames dwarf (df/df) mouse white adipose tissue after growth hormone (GH) and thyroxine (T4) treatment. For A‐D, wild‐type (N)n=10;df/dfn =9;df/dfGHn= 9;anddf/dfGH+T4n=8. ForE(FOXO3), Normal(N) n = 10;df/df n=9;df/dfGHn= 9;anddf/df GH+ T4n= 7. Groups that do not share a superscript show differences with statistical significance (p < 0.05). Both GH and GH + T4 treatment decreased PPAR-γ gene expression level in WAT of df/df mice (compared with df/df mice, p <0.05, p< 0.005 respectively). Moreover, animals in the df/df-GH + T4group had lower PPAR-γ expression than the df/df-GH group (p < 0.05) (Figure 8), PGC-1α mRNA levels were significantly increased in the WAT of Ames dwarf mice compared with N controls (p < 0.005).

STAT-1 and STAT-5a gene expression were increased in df/df as compared to N mice (p < 0.01) and normalized by the treatment with either GH or GH + T4 (both p < 0.05) (Figure 9),. The expression of STAT-5b showed upregulation in df/df mice when compared with N animals and decreases in response to both GH (p < 0.05) and combined GH + T4 therapy (p < 0.0005). The level of STAT-5b mRNA in df/df-GH + T4 mice was lower than in df/df-GH and N mice (p < 0.01 and p < 0.005, respectively). Similarly to the findings with STAT-5b, the expression of FOXO3 was elevated in df/df mice in comparison to N animals (p < 0.05), and significantly decreased by treatment with GH or GH + T4 in comparison to saline-injected df/df mice (p < 0.05, p < 0.0001). Adiponectin expression levels did not differ between N, df/df, and df/df-GH animals. However, df/df-GH + T4 mice had decreased WAT levels of adiponectin mRNA when compared with N, df/df, and df/df-GH counterparts (p< 0.01, p < 0.005, p < 0.05, respectively) (Figure 9).

## DISCUSSION

Treatment of juvenile Ames dwarf mice with a combination of GH and T4 for a period of 6 weeks did not affect their average longevity. Reduction of maximal longevity was detected only in females. These findings contrast with the ability of treatment with GH alone during the same period of development to reduce longevity in both female and male dwarfs from the same breeding colony ([6]; Hill Arum & Bartke, unpublished observations). This difference between the effects of treating juvenile Ames dwarf mice with GH + T4 vs. GH-only treatment was unexpected, particularly because both treatments produced a major increase in somatic growth. However, six weeks of treatment of juvenile Ames dwarfs with T4 alone did not impact longevity in our previous study [6], even though somatic growth was modestly increased. We have no explanation for a small but statistically significant increase in longevity in normal males treated with GH + T4 between the ages of 2 and 8 weeks or for the absence of a similar effect in females. Interpretation of findings in normal animals is complicated by the impact of injected GH and T4 on the functions of the somatotropic and the hypothalamic-pituitary-thyroid axis, which was not examined in the present study.

In an attempt to identify mechanisms that could have been responsible for the differential impact of the treatment with GH + T4 vs. GH alone on longevity, we have examined expression of a number of genes related to GH, IGF-1 and insulin signaling in the skeletal muscle and WAT, important target tissues for these hormones. The animals received GH or GH + T4 using treatment protocols identical to those employed in the longevity studies ([6, 43] and the present study) but were euthanized on the day following the last injection to compare the molecular alterations involved by these hormonal regimens.

There was no difference in bodyweight between GH and GH + T4 treated Ames dwarf mice at the time of sacrifice and the average body weights of df/df-GH and df/df-GH + T4 mice matched the values in the N control group. This differs from the results obtained by Louis et al. [43], who reported that treatment of male Ames dwarf mice with the same doses of GH as employed in the present study did not fully normalize their body weight. This difference may be related to the fact that during the pre- and peripubertal period, male mice grow more rapidly than females and reach higher body weight at the age of 8 weeks (approximately 25 vs. 19 g). Our failure to detect a reduction in blood glucose levels in dwarf as compared to normal mice represents another difference from the results of previous studies [5, 6, 45]. The present lack of difference in blood glucose level can be potentially explained by the very young age of the animals. Both df/df and N mice were at the age of 8 weeks and, at this age, N mice show no signs of insulin resistance. In our previous work, we have shown that the major differences in insulin resistance begin to appear when the mice are approximately 15 months old, which is most likely due to a decrease of insulin sensitivity during aging in N mice and better maintenance in long-living mutants [46].

Dominici and colleagues have shown that the alterations in insulin sensitivity in df/df mice can be tissue specific [47], which is consistent with our recent euglycemicclamp study of Ames dwarf mice [7]. In the present study IR, IRS-1, and PI3K gene expression in skeletal muscle showed no difference between dwarf and normal female mice. In WAT, IRS-1, PI3K, and Akt2 expression did differ between the N and df/df control groups and these differences were eliminated by treatment of the dwarfs with GH.

Combined treatment with GH + T4 suppressed IR expression in both WAT and muscle, and decreased IRS-1 expression to the levels measured in normal controls. Compared to the normal controls, IGF-1 mRNA levels in Ames dwarf mice were increased in SM and reduced in WAT, however, the observed reduction was not as severe as previously observed in hepatic tissue or in plasma. This different regulation of IGF-1 in examined tissues, especially in SM, represents GH-independent local regulation of IGF-1 in these tissues. It was previously reported that df/df mice have normal levels of IGF-1 protein and mRNA in the hippocampus when compared to N animals [48]. This and other data suggest that GH mainly regulates hepatic IGF-1 and its secretion to the circulation. However, other organs can produce this growth factor, but make only minor contributions to the circulating levels of IGF-1 [49-52]. GH-only treatment drastically increased local IGF-1 mRNA in both tissues while including T4 in the treatment regimen negated the effects of therapy with GH alone. Thus, female dwarf mice readily respond to treatment with GH and T4 in terms of somatic growth, but fail to exhibit major changes in the expression of genes related to insulin signaling in the skeletal muscle or in WAT in response to this treatment. Moreover, the results indicate that concurrent use of exogenous T4 can modify the effects of GH therapy on expression of numerous genes.

Reviewing the gene expression results, a common trend between both tissues examined in the present study is apparent. In the muscle, the mRNA levels for the genes: IR, PI3K, Akt2, GHR, IGF-1, GLUT4, STAT-1, STAT5b, and FOXO3, were lower in animals treated with GH + T4 than in those treated with GH alone. In WAT, the combined treatment decreased IR, IRS-1, PI3K, Akt2, PPAR-γ, GHR, IGF-1, GLUT4, STAT-5b, FOXO3, and adiponectin gene expression. In both SM and WAT, GH treatment alone failed to alter PPAR-γ gene expression, yet GH + T4 treatment led to PPAR-γ mRNA decreases in both tissues. However, some major differences were also apparent. For PGC-1α, both treatments produced expression levels mimicking those in the N control group in the WAT but not in the muscle. GLUT4 showed strikingly different effects of genotype and treatment in SM as compared to WAT.

Differential impact of GH + T4 treatment on longevity of males and females was not entirely unexpected. There are major sex differences in the pulsatile pattern of GH secretion in both humans and rodents [53-55].

Females have a higher baseline level of GH, as well as larger and more prolonged GH secretory periods compared to males. In males, GH is present at lower basal levels but maximal GH plasma concentrations are higher. Thus, the twice daily GH treatment regimen used in the present study may have more closely mimicked the natural GH secretion patterns of males, reaching higher plasma peak concentrations for a shorter duration [53, 56, 57]. In this study, it is apparent that there are some sex-related differences in the effects of this hormonal therapy, as seen by the different effects on lifespan. Interpreting the changes in gene expression, it must be mentioned that changes in the mRNA levels do not always correlate to changes in translation and protein expression. Additional, more extensive studies would be needed to evaluate the levels and activation/deactivation of different signaling proteins to determine what steps of the pathway are being regulated by these hormones in the different tissues. These additional steps are necessary as we reported that df/df mice have higher whole body insulin sensitivity with corresponding enhanced glucose uptake by WAT and SM [7]. However, the present results based on our measurements of mRNA levels indicate that the regulation of this signal may differ in these two major insulin dependent organs. These differences may have been related to the timing of tissue collection or to compensatory adjustments in the insulin signaling pathway in experiments involving acute stimulation by insulin.

Furthermore, it would be pertinent to analyze the effects of hormonal treatment on these molecular signaling pathways at different stages of life and at different times after completion of the hormonal therapy. These future longitudinal experiments would allow the detection of persistent or permanent changes that GH or GH + T4 may induce during development. This could identify genes or pathways that could affect our lifespan or healthspan via developmental programming.

In conclusion, combining T4 treatment with GH therapy suppressed mRNA expression for several genes including GLUT4 and IGF-1compared to GH injections alone. Thyroxine suppressed the expression of genes involved in the GH signaling pathway in the muscle, including the GHR gene, thus counteracting some of the effects of GH replacement in the GH + T4 group. It is tempting to speculate that these actions of exogenous T4 may have been responsible for eliminating the impact of concomitant GH therapy on longevity, or at least may have contributed to the differential impact of GH + T4 vs. GH alone on lifespan. It is also possible that the impact of thyroid hormones on the development and maturation of the central nervous system and other organs results in shortening or a temporal shift of the period when GH can produce effects (most likely epigenetic) that alter the course of aging and modulate longevity. By elucidating the effects of GH and T4 replacement therapy on the insulin signaling pathway of GH and TSH deficient Ames dwarf mice, we hope to enhance understanding of the interactions between these hormones in the physiological control of whole body insulin sensitivity, aging and longevity. Progress in this area could support development of new, safe ways to improve glucose homeostasis in type 2 diabetic patients and in the elderly, promoting better health and quality of life for the future.

## MATERIALS AND METHODS

### Animals

In a breeding colony, phenotypically normal heterozygous (N) females were mated with homozygous dwarf (df/df) mutant males to produce the Ames dwarf and N mice. The mice were housed under light- and temperature-controlled conditions, with a 12-hour light and 12-hour dark cycle and the temperature maintained at 20-23°C. Nutritionally balanced food was provided ad libitum (Rodent Laboratory Chow 5001: 23.4% protein, 4.5% fat, 5.8% crude fiber; LabDiet PMI Feeds, Inc., St Louis, MO). The animal studies were completed at Southern Illinois University in Springfield Illinois following SIU Laboratory Animal Care Committee approval [42, 43].

### Effects of treatment of juvenile normal and ames dwarf mice with GH plus T4 on longevity

Exogenous T4 stimulates somatic growth of Ames dwarf mice and enhances the growth-promoting effects of GH [6, 43]. Treatment of juvenile Ames dwarfs with GH reduces their longevity, while treatment with T4 does not [6]. In view of these observations, we decided to examine the impact of early-life treatment with a combination of GH and T4 on the longevity of normal and Ames dwarf mice. Starting at the age of 2 weeks, female and male normal and Ames dwarf mice were injected twice daily with saline or with porcine GH (twice daily) plus L-thyroxine (three times per week) (See details later in section). The treatments were continued for six weeks. There were 24 animals per genotype/sex/treatment group (i.e. 24 N male control mice, 24 N male GH + T4, 24 df/df male control, 24 df/df male GH + T4, 24 N female control mice, 24 N female GH + T4, 24 df/df female control and 24 df/df female GH + T4). The animals were checked daily for survival. The dates of death were recorded. In some instances, animals that appeared moribund and unlikely to survive longer than another 24-48 hours were euthanized and the date of euthanasia was used for longevity determination.

### Comparison of the effects of treatment with GH alone vs. GH plus T4

At the age of 2 weeks, phenotypically normal and Ames dwarf female mice were divided into four experimental groups, including: (i) a control group of normal heterozygous (N) mice injected with 0.9% saline, (ii) Ames dwarfs (df/df) treated with 0.9% saline, (iii) Ames dwarfs treated with porcine GH (df/df-GH), and Ames dwarfs treated with GH and T4 (df/df-GH + T4). There were 8-10 animals in each group. Hormone treatment started at 2 weeks of age and continued for 6 weeks. Twenty-four hours after the last injection and after an overnight fast, the mice were sacrificed by isoflurane anesthesia followed by cervical dislocation. Fasting blood glucose levels were measured before sacrifice in blood collected from the tail vein using the OneTouch Ultra glucose meter (Life Scan, Inc. Milpitas, CA). Tissues were harvested, immediately frozen on dry ice and stored at −80°C until analysis [42, 43].

### Treatment

For preparation of the GH, porcine GH (Alpharma, Victoria, Australia) was dissolved in 0.1M NaHCO3 for stability, then 0.9% saline was added to make a single injection concentration of 21μg/50μL for the average animal body weight of 7g (3μg/g of body weight); pH was adjusted from 8.3 to 7.8. GH injections were performed twice a day on weekdays with each subcutaneous injection administering half the daily dose (~3μg/g); the first injection was ~9am and the second was ~4pm (~6μg/g/day total GH treatment). On weekends, only one injection was performed with the full daily dosage (~6μg/g/day).

For the preparation of the T4, L-thyroxine (Sigma, St Louis, MO) was dissolved in a 0.9% saline solution with a pH of 7.8. It was administered three times per week (Monday, Wednesday, and Friday) via subcutaneous injections (0.1μg/g body weight; 0.7 μg/50μL dose) and administered in conjunction with the respective morning GH doses, albeit delivered in separate injections.

The doses were selected on the basis of previous studies in Ames dwarf mice [42, 43].

### Real-time PCR

Hind leg skeletal muscle and periovarian white adipose tissue (WAT) RNA were used for the analysis of the expression levels of the following genes: IR, IRS-1, PI3K, Akt2, PPAR-γ, PGC-1α, GHR, IGF-1, GLUT4, STAT-1, STAT-5a, STAT-5b, Adiponectin, and FOXO3. First, total mRNA was extracted from the sample tissue using the Qiagen miRNeasy Mini Kit. Approximately 75mg of skeletal muscle tissue was cut from each sample, and then placed in a 1.5mL safe-lock tube with 1mm Zirconium oxide beads and 700μL of QIAzol Lysis Reagent. Samples were homogenized in a bullet blender at speed 10 for 2 minute periods, and then cooled on wet ice for 2 minutes. This process of blending and cooling was repeated 3 times per sample, or until well homogenized. From here, the Quick-start Protocol included in the kit was followed as outlined by Qiagen, including the DNase digest steps. The result of using the kit was approximately 40μL of purified mRNA in nuclease free water (NF H2O). Approximate total RNA concentration was determined for normalization using agarose gel electrophoresis as well as Nanodrop procedure with the Epoch Gen5 Plate Reader (see appendix for procedure details). RNA extraction from white adipose tissue was performed in the same way, except 50-60mg of sample tissue was used, 0.5mm Zinc Oxide beads were used, and the bullet blender was set at speed 8 for 3 min.

Following the RNA extraction procedure, the cDNA synthesis was performed using the Bio-Rad iScript kit. After combining the master-mix (5x iScript reaction mix and iScript reverse transcriptase) with mRNA templates (derived from the tissue samples) in PCR tubes according to protocol, the reaction mix samples were placed into a Bio-Rad MJ Mini Personal Thermal Cycler. The machine conditions were set at 25°C for 5 min, 42°C for 30 min, and 85°C for 5 min, and then held at 4°C when finished. This led to cDNA samples in 20μL of NF H2O; NF H2O was added to each sample to dilute them and narrow the range of B2M cycle threshold (CT) values with RT-PCR (see below). The cDNA samples were stored at 4°C until analysis (if there was a longer period of time between cDNA synthesis and RT-PCR analysis, the cDNA samples were frozen at −20°C).

This cDNA was used for RT-PCR analysis. 2μL of cDNA was used per sample in a 96 well PCR plate. The forward and reverse primers for the gene of interest were prepared with SYBR Green and NF H2O in a master mix according to Applied Biosystems protocol, then added to the 96 well plate containing the cDNA samples for a total of 20μL per well. For PCR protocol and table of primers used, see appendix.

To normalize the RT-PCR data, beta 2-Microglobulin (B2M) was selected as the housekeeping gene. When CT values had a maximum range of 3 between all samples, gene analysis began. The following equation was used for calculating relative expression: 2^A-B^/2^C-D^ (A = CT value of the gene of interest in the first control sample, B = CT value of the gene of interest in each sample, C = CT value of B2M in the first control sample, D = CT value of B2M in each sample). This led to the first control sample having a relative expression of 1, and all other samples were calculated in relation to this first sample. The results of the normal (N) group were averaged, and all other results were divided by this average to obtain the fold change of expression of the genes of interest compared to this control group [43].

### Statistical analysis – longevity

Overall survival was tested by log-rank test, using GraphPad Prism 5. Maximal survivorship was evaluated as previously described [44].

### Statistical analysis – RT PCR

Microsoft Excel and GraphPad Prism5 software was used to calculate the raw data. Statistics were calculated on Prism5 via one-tailed P-values for unpaired t tests. Differences between groups that generated a p-value less than 0.05 were considered statistically significant.
